# Electrosynthesis and electrochemistry

**DOI:** 10.3762/bjoc.11.105

**Published:** 2015-06-02

**Authors:** Siegfried R Waldvogel

**Affiliations:** 1 Institute for Organic Chemistry, Johannes Gutenberg University Mainz, Duesbergweg 10–14, 55128 Mainz, Germany

**Keywords:** chemical method, electrochemistry, electrosynthesis, sustainability

Since the pioneering work of Kolbe, electrochemistry and electrosynthetic methods have been a part of the repertoire of the organic synthesis toolbox [1–2]. In general, only electrons are employed as reagents or the reagents are electrochemically regenerated. Consequently, waste can be avoided, and limited resources can be used in a careful and economic manner. Because alternative reaction pathways are employed by electrosynthetic methods, scarce and toxic elements can be replaced or are not required at all [3]. Moreover, in the foreseeable future regenerative sources of electricity, for example, photovoltaics and wind power, will provide a surplus of electricity as the current unsteady supply does not match the demand. Thus, the use of abundant electric power in electrosynthetic processes seems to be rational because high valorisation can be expected. Therefore, electrosynthesis fulfils all requirements for “green chemistry” [4]. When changing feed stocks and natural resources begin to play a more crucial role, electrosynthetic methodologies will not only be of ecological interest but also of economic significance. Unfortunately, the research in the past two decades was understated and considered as a niche methodology by the synthetic community. In addition, electrochemistry is mostly taught by physical chemists, which seems to create a natural barrier to preparative organic applications. However, the systematic use of cationic species as intermediates to avoid over-oxidation establishes new ways for functionalization of substrates and paves the way to novel synthetic tools [5–8].

Recently, a renaissance of electro-organic methods occurred in several fields, including the construction of rather complex molecules (e.g., natural products) [9]. Not only is the construction of biologically active molecules of interest but also the anodic degradation of drug-like molecules. Such electro-oxidative treatment generates potential metabolites that can be then biologically studied [10]. The combination of electrosynthesis with other powerful techniques, such as ultrasonic treatment and flow microcells, will push the electrosynthetic applications beyond current limits [11].

In addition, remarkable breakthroughs have been achieved regarding electrodes and electrolytes, which allow for expansion of the electrochemical window and/or novel reaction pathways. This leads to new electro-organic concepts and further applications for a sustainable synthetic methodology. The contributions within this Thematic Series demonstrate the broad use of electrosynthesis and represent a snapshot of this current and vividly developing field. I am convinced that electro-organic synthesis is an emerging field and that this issue will stimulate the reader to employ electrochemical methods in their own field.

Siegfried R. Waldvogel

Mainz, April 2015
